# Calculation of 3D genome structures for comparison of chromosome conformation capture experiments with microscopy: An evaluation of single-cell Hi-C protocols

**DOI:** 10.1080/19491034.2018.1438799

**Published:** 2018-03-08

**Authors:** David Lando, Tim J. Stevens, Srinjan Basu, Ernest D. Laue

**Affiliations:** a Department of Biochemistry, University of Cambridge, Cambridge, United Kingdom; b MRC Laboratory of Molecular Biology, Francis Crick Avenue, Cambridge Biomedical Campus, Cambridge, United Kingdom

**Keywords:** Chromosome conformation capture, Hi-C, fluorescence microscopy, genome structure, nuclear architecture

## Abstract

Single-cell chromosome conformation capture approaches are revealing the extent of cell-to-cell variability in the organization and packaging of genomes. These single-cell methods, unlike their multi-cell counterparts, allow straightforward computation of realistic chromosome conformations that may be compared and combined with other, independent, techniques to study 3D structure. Here we discuss how single-cell Hi-C and subsequent 3D genome structure determination allows comparison with data from microscopy. We then carry out a systematic evaluation of recently published single-cell Hi-C datasets to establish a computational approach for the evaluation of single-cell Hi-C protocols. We show that the calculation of genome structures provides a useful tool for assessing the quality of single-cell Hi-C data because it requires a self-consistent network of interactions, relating to the underlying 3D conformation, with few errors, as well as sufficient longer-range *cis*- and *trans-*chromosomal contacts.

## Introduction

Chromosome conformation capture (3C) is a biochemical technique where contact frequencies between two particular genomic sequences in a population of cells are quantified after restriction enzyme digestion and proximity-based DNA ligation [1]. The simple and elegant idea that digestion and re-ligation of chromatin could be used to measure contact frequencies has meant that 3C (one to one) and its many related techniques (4C: one to all, 5C: many to many, and Hi-C: all to all) have now been widely utilized to study genome structure. Together, they have revealed a hierarchical organization where above the megabase scale the genome is partitioned into large regions enriched in transcriptionally active or repressed genes, referred to as the A and B compartments [2]. At scales below a megabase, the genome has smaller, self-associating regions of chromatin called “topological-associated” domains that are supported by DNA loops mediated by the Cohesin complex (for a recent review see Denker & de Laat [3]).

Our initial attempts to calculate 3D chromosome/genome structures using population data (millions of cells) showed that there were many conflicting contacts, suggesting that there was no dominant, underlying 3D conformation and hence considerable variation in structure from cell-to-cell. Thus, it was not initially clear to what degree the genomic features discussed above may exist in single cells. In 2013 we were able to calculate the first structures of the X chromosome from diploid mouse T_H_1 cells using data from a new single-cell Hi-C experiment, which introduced in-nucleus digestion [4]. Whilst the number of contacts from a single T cell (several thousand) was substantially lower than canonical, population Hi-C data (tens to hundreds of millions of contacts) it was immediately clear that there was indeed a high degree of cell-to-cell variability in chromosome structure.

**Figure 1.**
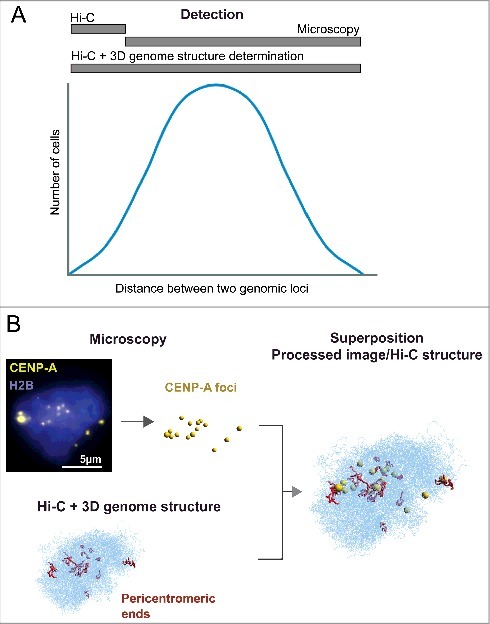
3D genome structures calculated from single cell Hi-C data can detect the entire range of distances between two pairs of loci for comparison with microscopy images. (A) In a population of cells 3C based techniques such as Hi-C can only detect distances between two genomic loci when they are very close together. Due to the resolution limit imposed by the diffraction of light (∼200 nm), close distances between two loci cannot be detected accurately by microscopy. 3D genome structures calculated from single cell Hi-C data, however, allow one to detect the distance between two loci regardless of whether they are very close together or far apart. (B) A superposition of CENP-A foci from a microscopy image with a 3D genome structure calculated from Hi-C data from the same imaged cell. Pericentromeric ends of the chromosomes are labelled in red. CENP-A foci from the microscopy image depicted as a point cloud are colored yellow. The scale bar in the microscopy image is 5 µm. Figure adapted from Stevens et al. [9].

More recently, differences have been reported between contact frequencies obtained from population 5C data and the corresponding distance measurements obtained with DNA Fluorescence In-Situ Hybridization (FISH) experiments, leading to concerns about the validity of chromosome conformation capture data [5]. However, these discrepancies are expected to arise because population 3C-type experiments and microscope images detect quite different spatial features of chromatin [6,7]. In a 3C experiment carried out on a population of cells the interaction of two DNA loci is only detected in a subset of cells where the loci are sufficiently proximal to ligate, and direct contacts are not detected in cells where they are far apart. In contrast, due to the resolution limit associated with the diffraction of light, it is more difficult to accurately detect when two DNA loci are in close proximity (within a few hundreds of nm) to one another using microscopy (Fig. 1A). Although the problem of resolution in imaging can be mitigated using super-resolution methods [8], fluorescence microscopy only allows the investigation of a restricted set of labeled loci at one time.

By computing a complete 3D genome structure from single-cell Hi-C data we have shown [9] that it is possible to overcome the limitation of Hi-C, of only detecting DNA sequences in close spatial proximity, because we can now determine distances between loci regardless of whether they have direct contacts or not (see Fig. 1B where we study the distances between various pericentromeric loci in the computed Hi-C structure). A 3D image obtained by fluorescence microscopy and the 3D genome structure calculated from the Hi-C data can thus be directly compared, and, in a proof of concept, we superimposed images of the centromeric histone H3 variant CENP-A, which is localized at centromeres, with 3D genome structures [9] (Fig. 1B). Importantly, the combination of imaging and single-cell Hi-C experiments paves the way to directly relate a process being imaged with the underlying DNA sequence of the chromatin involved. There is also another significant benefit, however, because the combination of the two types of data (from the images and the Hi-C experiment), will in the future offer a route to dramatically improve the accuracy and precision of the 3D genome structures. As with other methods where structures are calculated from a network of only short-range restraints, e.g. protein structure determination by NMR spectroscopy using nuclear Overhauser effect-based restraints, when the calculations use only Hi-C contacts the larger scale 3D structure is not as well determined as the local structure. This can be overcome by combining global and long-range restraints determined using a different type of data. Therefore, in the longer term we can look forward to considerable improvements in the quality of the 3D genome structures, as we include image-based restraints in the structure calculations. To compute 3D genome structures from Hi-C data, however, the study of single cells is critical because it is only in this situation that the measured contact data represents a single underlying conformation. By contrast, in population Hi-C experiments the data represents a vast number of different conformations. Although probabilistic structural models can be derived from canonical, multi-cell, population Hi-C data [10], the models produced (either best-fit or deconvolved) cannot be expected to provide realistic 3D structures that could be found in any individual cell.

**Figure 2.**
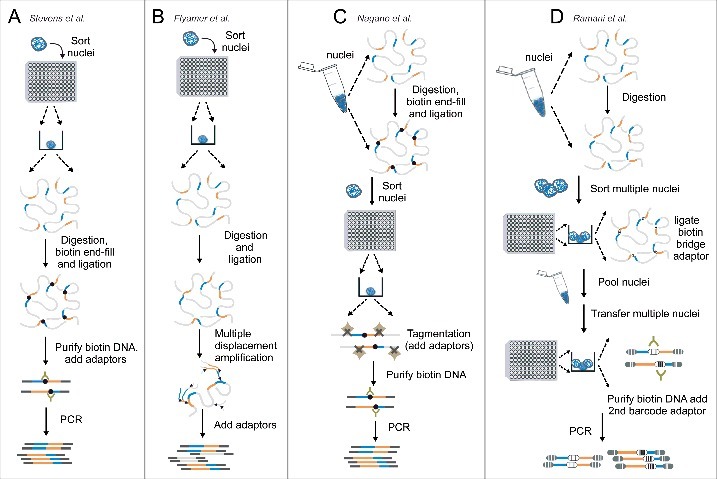
Schematic overview of the four different single-cell Hi-C protocols. Key steps in the procedures to make Hi-C libraries are highlighted for: (A) Stevens et al. [9], (B) Flyamer et al. [12], (C) Nagano et al. [13] and (D) Ramani et al. [14].

In this paper, we utilise the NucProcess computational pipeline [11] to evaluate the data provided by different single cell Hi-C protocols, all of which involve in-nucleus digestion [9,12−14]. In brief, both Stevens et al. [9] and Flyamer et al. [12] have developed protocols where all the Hi-C processing steps and library preparation are carried out on a single nucleus (Figs. 2A and B). This is necessary if one is to first image and then biochemically process the same single cell, because the biochemical steps increase the fluorescence background and degrade the image [11]. Flyamer et al. [12] additionally removed the biotin end-filling step that is used to purify ligated junctions away from non-ligated DNA, and they also replaced PCR with Multiple Displacement Amplification (MDA) when preparing the libraries for sequencing. Nagano et al. [13] took the original protocol, where the biochemical steps are carried out on a population of nuclei prior to the selection of individual nuclei for the preparation of libraries for sequencing [4], and replaced the multi-enzyme adaptor ligation steps with a single transposase reaction (Fig. 2C). This procedure, which was also tested with less success by Stevens et al. [9], significantly improves the efficiency of preparing the libraries. Finally, Ramani et al. [14] implemented an ingenious two-step barcoding system into the original protocol that vastly improves the throughput, allowing the processing of thousands of nuclei in a single experiment (Fig. 2D).

Here we carry out a systematic evaluation of the data sets generated by these different second-generation single-cell Hi-C protocols, with a focus on assessing which approaches can generate high quality contact data that can subsequently be used to compute realistic 3D genome structures, which may then be compared with data from different imaging methods.

## Results

### Comparative analysis of the sequence data

We used the NucProcess software (available at https://github.com/tjs23/nuc_processing) to process data sets generated using the four different protocols in a consistent, comparable way. NucProcess takes the paired-end sequence reads, maps the fragment ends back onto a reference genome sequence and then filters the paired fragments to determine which ones represent valid and useful contacts [9,11]. Read pairs that pass or fail any of the filtering steps are recorded, and these results are compiled into a report, but naturally only those pairs that pass all filters are used for the creation of the output contact maps. A summary of the key findings from NucProcess is presented in Table 1 and the results from the individual cells studied are available in the supplementary online data Table S1. The protocol developed by Flyamer et al. [12] generated the largest number of contacts, with an average yield of over 480,000 per cell. The protocols developed by both Nagano et al. [13] and Stevens et al. [9] yielded lower but similar numbers of between 70,000 and 80,000 contacts per cell (for equivalent G1 phase haploid data). Analysis of the largest 20 single-cell mouse data sets, of 500 processed from Ramani et al. [14], yielded an average of only 724 contacts per cell. The average input read pairs for the Ramani et al. [14] data was ∼26,000, around 100-fold less sequence data than any of the other three protocols (Table 1). By increasing the sequencing depth it is very likely that more contacts can be identified. However, due to the large number of cells processed and sequenced in each run (many thousands), it may not be easy to determine similar numbers of contacts to the other methods.

**Table 1.** Summary of the analysis of the sequence data for the different single cell Hi-C protocols.

	Read pairs		Filtered unique pairs		Identified contacts			
	Average input	% Unique	% Accepted	% Promiscuous	Average number	% Cis<10kb	% Cis>10kb	% Trans
Stevens	2,087,953	58.7	87.06	3.0	70,262	41.7	49.4	8.9
Flyamer	22,992,434	60.6	31.63	34.0	481,797	58.7	34.6	6.7
Nagano	8,346,671	54.3	69.67	3.8	77,584	42.2	51.2	6.6
Ramani	26426	33.1	97.67	0.1	724	33.4	48.3	18.3

A high number of contacts is often a good indicator of a useful DNA library for further analysis. However, the quality of the contact data, in terms of useful signal compared to both unwanted sequence pairs and illegitimate contacts, is also critical. Certainly some seemingly random, unwanted ‘noise’ contacts represent genome mapping problems (clearly seen in cells that are missing entire chromosomes [8) which could arise from sequencing errors and genetic drift from the reference genome. However, there are clearly also experimental sources of illegitimate contacts from spurious ligation events, which can potentially arise at various stages during the processing of the sample. For example, in the Stevens et al. [9] protocol it was found that spurious contacts occur when insufficient washing is carried out during the steps to add adaptor barcodes to the biotin purified ligated junctions [11] (Fig. 2A). Therefore, a more in-depth analysis of the processing results is needed to help identify and troubleshoot poor quality libraries. For example, the best single-cell data sets tend to contain a high proportion of reads (typically >50%) that map to unique locations in the reference genome sequence. The data sets from Flyamer et al. [12], Nagano et al. [13], and Stevens et al. [9] all passed this benchmark. In data sets produced using the protocol from Ramani et al. [14] an average of ∼33% of the reads mapped uniquely (Table 1). This indicates that the libraries from the Ramani et al. [14] protocol contain a lower proportion of useful contact data. This may be due to several reasons, such as species cross-over (given that a mixture of mouse and human cells were processed together) or over-amplification of primer or barcode sequences that can result from the handling of small amounts of DNA.

Useful libraries also contain a high proportion of read pairs with ligated junctions. We observe that when filtering the mapped reads (to identify valid contacts), the best single cell libraries tend to contain at least 50% of accepted read pairs. An analysis of the filtered read-pairs revealed that only ∼32% of the contacts were accepted as valid using the Flyamer et al. [12] protocol, showing that a large number of read pairs lacked a ligation junction between two desired primary restriction enzyme (RE1) sites. Subsequent analysis of the filtering results showed that this protocol generates a large proportion, nearly 50 to 60% of read pairs, that map to either the same or adjacent RE1 restriction fragments (see the report on the Flyamer et al. [12] data in **Table S1**). Mapping of read pairs to the same RE1 fragment indicates that a read pair does not span a ligated junction. Mapping of read pairs to adjacent RE1 sites may or may not represent a ligation event, but either way they do not represent a useful contact. There is no specific purification step for ligated junctions in the Flyamer et al. [12] protocol, and this may explain why so many reads map back to close positions in the reference genome and why the libraries need to be sequenced to a much greater depth than the other three protocols (Table 1). In addition, the filtering of the contacts revealed that this protocol produced an average of 34% promiscuous contact pairs, 10-fold more than any of the other protocols (Table 1). A promiscuous contact refers to the situation when one of the restriction fragment ends occurs in more sequence pairs than would be expected, given the ploidy of the cell. On a large scale, this may arise either when multiple cells are accidently processed together, or when the processing of nuclei generates spurious read pairs between genomic loci that are not genuine contacts.

**Figure 3.**
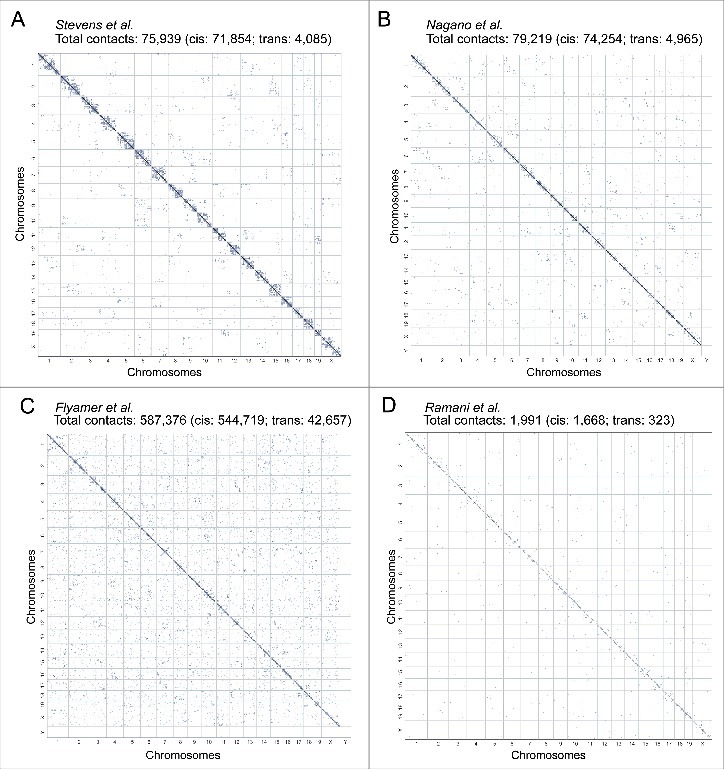
Genome wide contact maps from the different single-cell Hi-C protocols. The total identified contacts were plotted as an all-vs-all matrix of genomic positions for: (A) Cell 6 from Stevens et al. [9]; (B) NXT-103 from Nagano et al. [13]; (C) Zygote Mat 71 from Flyamer et al. [12] and (D) MMHiC_TGGAGAGG_ACAGACTG from Ramani et al. [14]. Each blue pixel represents contacts mapped within a bin size of 5 × 5 Mb. The total number of contacts, along with the breakdown between *Cis* and *Trans* within and between chromosomes, are shown above each map.

In addition to the reports, to provide another way to assess the quality of the single cell Hi-C libraries, NucProcess allows one to plot the filtered contacts on a genome-wide map (Fig. 3). A good contact map contains a large number of long-range *cis*-chromosomal interactions near the diagonal of the map, with discrete clusters of *trans*-chromosomal interactions between distinct pairs of chromosomes; a feature resulting from the chromosomes occupying distinct nuclear territories. Visual inspection of the contact maps illustrating both the Nagano et al. [13] and Stevens et al. [9] data reveals significant numbers of long-range *cis*-chromosomal contacts and obvious clusters of *trans*-chromosomal contacts between a restricted number of pairs of chromosomes (Figs. 3A and 3B). Contact maps illustrating the Flyamer et al. [12] data also show enrichment of long-range *cis*- and *trans*-chromosomal contacts (Fig. 3C), but they reveal a noticeable number of seemingly random contacts with much less clustering between particular chromosomes. This higher level of random contacts suggests that the Flyamer et al. [12] protocol is generating proportionately more spurious contacts than either the Nagano et al. [13] or Stevens et al. [9] protocols. The low number of contacts identified per cell using the Ramani et al. [14] protocol results in contact maps that are too sparse to see any *trans*-chromosomal clustering, although there is evidence of an enrichment in long-range *cis*-contacts. This suggests that the main problem when using this protocol for the analysis of individual cells is a lack of data (Fig. 3D).

### Differences in genomic contact sequence separation

**Figure 4.**
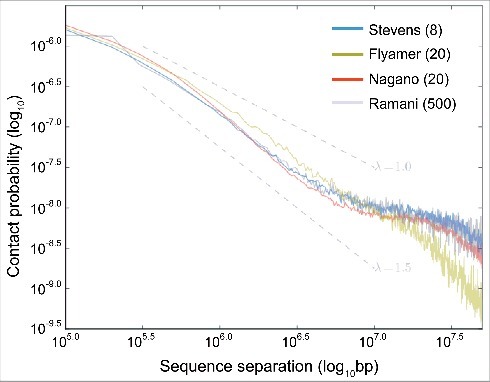
Analysis of contact probability with sequence separation for data produced with each of the single-cell Hi-C protocols. Log-scale plots of contact probability against sequence separation for combined single-cell Hi-C contact data, binned at 100 kb. The number of single cells analysed for each protocol are shown in brackets. The slopes for a power law relationship (Contact probability for sequence separationα) in which α is either −1.0 (fractal globule polymer) or −1.5 (equilibrium globule polymer) are indicated as grey dashed lines.

In the original 3C experiments, examination of the data revealed that the contact probability between two loci decreases as a function of genomic distance. This rate of decrease supported a polymer model for chromosome folding [1]. More recently Hi-C data has suggested that chromosome structure can be described by a non-equilibrium, fractal-like globule [2]. To examine whether similar distributions of contact probability are observed using the new single-cell protocols we pooled the contact data sets for each protocol, which were carefully selected (where possible) to be haploid and to avoid condensed, mitotic nuclei. For each set of data we then plotted the contact probability against sequence separation (Fig. 4). While the overall slope of the sequence separation curve for the four protocols was similar to that measured previously by other 3C based techniques, the results did reveal some differences. Some of these are minor. For example, with the Nagano et al. [13] protocol shorter range contacts (separations from 0.1 to 1.0 Mb; 10^5.0^ to 10^6.0^ bp) are slightly enriched and longer-range contacts (separations >3.0 Mb; 10^6.5^ bp) are slightly depleted, when compared with the Ramani et al. [14] and Stevens et al. [9] datasets. These differences clearly arise from the way the nuclei are processed. Indeed, both Nagano et al. [13] and Stevens et al. [9] used the same haploid mouse ES cell line in their studies and so the slight enrichment of shorter over longer range contacts detected by Nagano et al. [13] likely stems from the protocols. Here, the use of a transposase enzyme to add the adaptors by Nagano et al. [13], or variations in the way in which the in-nucleus digestion, end-fill and ligation steps are performed may explain these differences. The most striking difference, however, was seen with the (MDA based) Flyamer et al. [12] data. Compared to the other three protocols the Flyamer et al. [12] protocol yields notably fewer long-range *cis*-contacts with separations greater than 10 Mb (10^7.0^ bp) and consequently the data is enriched with contacts having separations less than this.

### Comparison of 3D genome structures

As discussed, a major advantage of single-cell Hi-C, compared to the multi-cell population equivalent, is that the contacts between genomic sites can be used as restraints in calculations to compute 3D genome structures, e.g. involving simulated annealing of a particle-on-a-string representation of chromosome structure. The simulated annealing approach to chromosome structure calculation is discussed in detail in the supporting material for previous publications [4,9]. However, the general notion is that mildly repulsive particles (which cannot superimpose) are connected into chromosome backbones and the single-cell Hi-C contacts, represented by short distance restraints, pull contacting particles together during the calculation so that they touch. The interpretation of single cell Hi-C contacts as particles within a close, touching distance is very straightforward compared to population Hi-C. Repeat calculations from different random starting coordinates generates a family of alternative models for each cell, but with sufficient data (> 60,000 contacts, of which > 5% are *trans*) the expectation is that the models will be highly similar and thus represent a single folded genome structure, with unmappable (highly repetitive) sequence regions differing the most.

**Figure 5.**
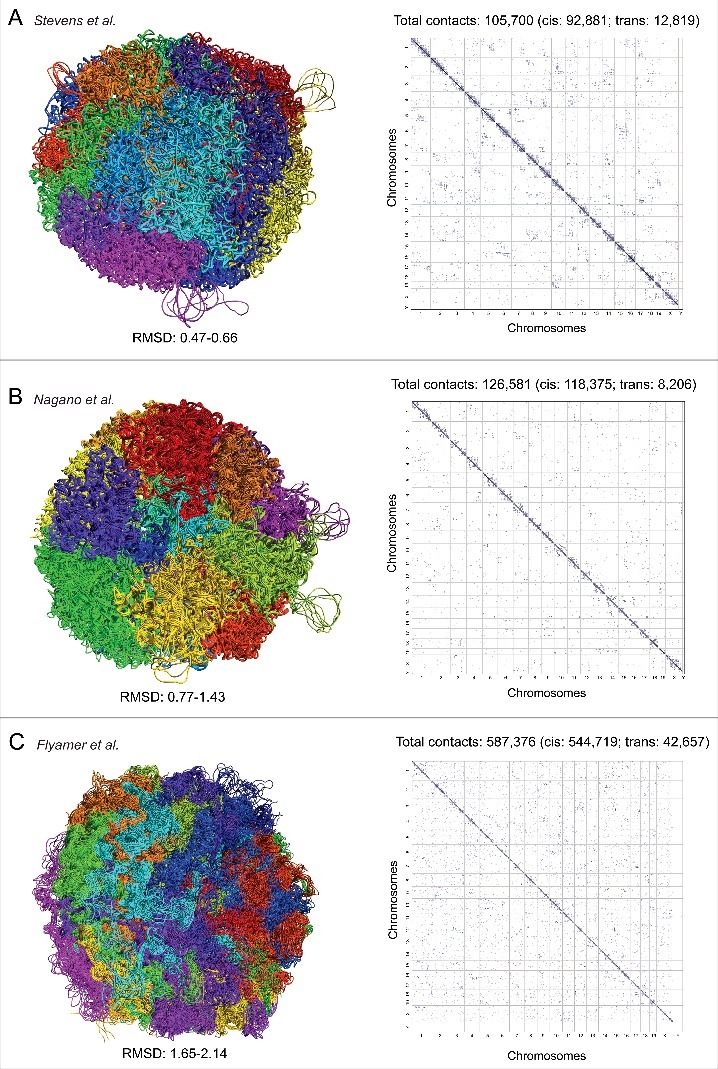
Comparison of 3D genome structures calculated from different single-cell Hi-C contact data-sets. Shown are five superimposed structures derived from repeat calculations using 100 kb particles, along with the corresponding contact map (see Fig. 3 for details), for: (A) Cell 1 from Stevens et al. [9]; (B) NXT-117 from Nagano et al. [13]; and (C) Zygote Pat 63 from Flyamer et al. [12]. Contacts represent the number of contacts used to generate the structural models after removing any contacts that are not supported by others within a genomic window of 2 Mb (to reduce noise). The RMSD values are represented as a range derived from the 100 kb all-particle values between all the model pairs. Each of the 20 chromosomes (Chr1-19 & ChrX) is coloured differently.

The resulting 3D genome structures may then be directly compared and superposed with 3D images from fluorescence microscopy [9]. They can also be inspected to identify where in the nuclear volume (within the resolution of the model) all loci lie relative to one another. In turn, this allows many types of analyses, including those that look at distances, spatial clustering, backbone conformation etc [9,15]. Also, computing structures provides a means to assess single-cell Hi-C data and remove spurious contacts because it readily identifies those that are inconsistent with the otherwise self-consistent 3D network. To compare the applicability of the protocols to produce data for 3D whole-genome structure calculations, we took a sample data set produced by each of the three protocols producing the highest numbers of contacts (from Flyamer et al. [12]; Nagano et al. [13] and Stevens et al. [9]) that contained well defined clusters of *trans*-chromosomal contacts (Fig. 5). We then calculated 10 independent models, starting from different random starting coordinates, for each data set. [Currently, the data sets produced by Ramani et al. [14] are too sparse to contain sufficient long-range *cis* and/or *trans*-chromosomal contacts to compute structures.]

For all the test datasets we employed the NucDynamics software (available at https://github.com/tjs23/nuc_dynamics) in the same way, using its default parameters, and in Fig. 5 an overlay of five models (chosen at random) of each computed whole genome structure is shown. In these calculations each particle represents 100 kb of chromatin. By calculating multiple models, it is possible to gauge the precision of the computed structures – in other words how well determined they are by the experimental data – by measuring the root-mean-square deviation (RMSD) value between the different models. While the dataset from Stevens et al. [9] contained the fewest contacts, analysis of the RMSD values revealed that structure had the lowest range of model-model RMSD values: 0.47 to 0.66 particle radii. This likely results from the increased proportion of longer range (>10 Mb) contacts produced by this protocol compared with the data from Nagano et al. [13]. Notably, the structure computed from the Flyamer et al. [12] data had the highest RMSD values (1.65-2.14 radii), despite having five times more contacts than the data sets from either Nagano et al. [13] or Stevens et al. [9] This difference in precision, which likely stems from a relative paucity of longer-range contacts combined with a large number of spurious contacts, can be clearly seen when viewing the structures of multiple models of a particular chromosome (**Fig. S1**). In addition, whilst the chromosomes in the structures computed from either the Nagano et al. [13] or Stevens et al. [9] data form discrete (yet intermingling) territories, this is not the case for all the chromosomes computed from the Flyamer et al. [12] data. Here, chromosomes are often entangled and some regions adopt extended conformations (**Fig. S1**). Further work is necessary to understand how much of this is due to the different cell-type rather than problems with the data.

## Discussion

In this study we have implemented a computational pipeline for the evaluation of single-cell Hi-C data. We also wanted to carry out a first comparative analysis of the four recently published protocols, with a view to evaluating which may be most useful for calculating 3D genome structures. Ultimately, the aim is to produce the best quality chromatin contact data sets that can then be used to compute genome structures at the finest possible resolution. We find that three of the four protocols by Stevens et al. [9], Flyamer et al. [12] and Nagano et al. [13] generate sufficient numbers of useful contacts to compute genome structures at 100 kb resolution. The fourth protocol by Ramani et al. [14] allows the study of many more cells but currently yields too few contacts for 3D genome structure determination, although it is of course useful as a low-input Hi-C variant for statistical analyses. The protocols by Stevens et al. [9] and Nagano et al. [13] yielded on average a comparable number of genomic contacts, 70,000 and 77,000 respectively (Table 1), to produced genome structures with good precision (RMSD < 1.5 radii) at 100 kb resolution. This yield represents an average recovery of 2.3-2.5% of the total possible ligation junctions, and it is likely that these two protocols have yet to reach their contact capture limit. The incorporation of a large bulky biotin dATP nucleotide at ligated junctions, which has been reported to be inefficient [16], may be a reason for the low yield in these two protocols. Indeed, the protocol by Flyamer et al. [12] which omits biotin labelling generated on average over 6 fold more contacts (481,000) than the protocols developed by either Nagano et al. [13] or Stevens et al. [9] (Table 1). However, we discovered that the computed structures from these data sets are of poorer precision than those from either the Nagano et al. [13] or Stevens et al. [9] data (Fig. 5). This probably results from a smaller proportion of long-range contacts and the presence of illegitimate contacts – the latter being identifiable as uniformly dispersed contacts in the contact map. Some differences in contact probability can naturally be expected due to differences in cell type (where chromosome folding may be different). The Flyamer [12] protocol was applied to mouse oocyte and zygote samples, which are both found in the early stages of development, whereas Stevens et al. [9] and Nagano et al. [13] studied mouse ES cells. A recent population Hi-C study carried out with mouse oocytes reported that the contact maps contain less well-defined TADs and compartments compared to ES cells [17]. Therefore, the different cell types could be responsible for some of the differences in the distribution of Hi-C contacts. However, the differences in the levels of random, unclustered contacts seem to stem from the Flyamer et al. [12] protocol. Our analysis of the processed sequence data reveals substantially more promiscuous reads (i.e. reads that are involved in more contacts than is formally possible for a single cell), compared to the data from either Nagano et al. [13] or Stevens et al. [9] (Table 1). This suggests that the Flyamer et al. [12] protocol generates more random, illegitimate contacts. In this context it is noteworthy that the Flyamer et al. [12] protocol is the only one to use the strand displacement Phi 29 DNA polymerase for library amplification, whereas all the other protocols use a thermally stable DNA polymerase and PCR for DNA amplification. It has been reported that multiple displacement amplification can generate chimeric DNA rearrangements between two different DNA molecules complicating genome assembly [18]. Analysis of these types of rearrangement have revealed that they probably occur when the displaced polymerase 3′-termini are freed and prime to nearby displaced 5′-strands to create a chimeric molecule [19]. An increase in such chimeric products may help explain the large number of illegitimate *trans*-chromosomal contacts observed in the genome wide contact maps obtained with this protocol. To overcome these problems an alternative strategy might be to employ a different single cell WGA method such as linear amplification via transposon insertion (LIANTI) [20]. This procedure does not use a strand displacement polymerase, but instead combines Tn5 transposition and T7 in-vitro transcription to linearly amplify DNA. Interestingly, the protocol developed by Nagano et al. [13] successfully employed the Tn5 transposase to introduce primer sequences for PCR amplification, suggesting that Hi-C ligated DNA is permissive to DNA insertion by transposition and that a LIANTI approach could potentially be used to make Hi-C libraries from single cells.

In conclusion, single-cell genomic methods are developing rapidly and we hope that the computational pipeline described here will provide a useful tool to suggest how the current protocols might be improved. They should also be useful to assess new single-cell Hi-C protocols as they are developed and to troubleshoot experiments as these protocols are utilized in practice.

## Materials and methods

### Software

Unless otherwise stated, all Python scripts for processing and analysis are available at: https://github.com/tjs23/nuc_processing and https://github.com/tjs23/nuc_dynamics.

### Single-cell Hi-C sequence data

Sequencing data corresponding to the four single-cell Hi-C protocols were downloaded from the Gene Expression Omnibus (GEO) entries with the following accession codes: GSE80006^12^, GSE94489^13^, GSE84920^14^ and GSE80280^9^. Corresponding SRA entries, as listed in **Table S1** were then used as the source of sequence data for single-cell samples (haploid where possible). The SRA files were downloaded and automatically converted into FASTQ format file using the “*fastq-dump –split-files*” command from the NCBI SRA toolkit.

The various sequence data sets were then processed, as required, to extract sequences for separate, individual cells. The SRA data sets from Flyamer et al. [12] and Stevens et al. [9] were already separated into individual cells and so these reads could be used directly after downloading in FASTQ format.

Data from Nagano et al. [13] was in the form of separate cell-barcode and main-read sequence FASTQ files. Corresponding barcode and main-read files, representing the same paired-end group for the sequenced library (i.e. R1 or R2), were spliced together using the splice_fastqs.py script and then de-multiplexed, according to the barcode sequences, into separated paired FASTQ files for each cell using the following script and command line options:

python split_fastq_barcodes.py comb_seqs.fq -b barcodes.txt -s 8

Data for the massively-multiplex method of Ramani et al. [14] was initially processed using the first stages of the method described at https://github.com/VRam142/combinatorialHiC and its corresponding Python scripts. The SigPrep (https://github.com/jstjohn/SeqPrep) command was used to remove adapter sequences and the inline_splitter.py script was used to trim and record outer barcodes for the reads:*SeqPrep -A AGATCGGAAGAGCGATCGG -B AGATCGGAAGAGCGTCGTG -f in_r1.fq -r in_r2.fq -1 ad_clip_r1.fq -2 ad_clip_r2.fq*

python inline_splitter.py ad_clip_r1.fq ad_clip_r2.fq outer_barcodes.txt split_r1.fq split_r2.fq 2> splitting_stats.html

The clipped files were then passed on to the analyze_scDHC_V2design.py script to find and remove inner barcode sequences:

python analyze_scDHC_V2design.py inner_barcodes.txt split_r1.fq split_r2.fq bc_clip_r1.fq bc_clip_r2.fq > bc_assoc.txt

Next, a custom written Python script “*split_mmhic.py*” was used to separate the files into individual single-cell FASTQ files according to their barcode associations – the original paper mapped reads from all cells together. The largest of these were then selected for further processing analysis. These single-cell datasets were filtered according to species (i.e. selecting mouse rather than human) at a later stage. During the sequence processing mapping human reads to the mouse genome gives detectably poor results.

### Sequence processing and analysis

All genome sequence mapping, processing and analysis was performed using the NucProcess software using the same parameters where possible, but with key adjustments appropriate to each data-set's protocol, e.g. accounting for whether fragments were released randomly or using a second restriction enzyme. In each case, the inputs were paired-end FASTQ format read files and the outputs were filtered contact lists, contact maps and statistical reports, all for each cell separately. It is notable that this procedure performs somewhat more stringent checks for aberrant contacts than was described by Flyamer et al. [12], where a relatively simple binning and de-duplication procedure was employed.

NucProcess, as described in Stevens et al. [9] and Lando et al. [11], performed the following steps: 1) Reads that go through a ligation junction were clipped at the junction to remove any unnatural sequence that should not be mapped to the reference genome. 2) The clipped reads for each molecule end (R1 or R2) were mapped separately to the mouse genome build (GRCm38/mm10) using the Bowtie2 program {PMID:19261174} [21] with options corresponding to ‘*bowtie2 –very-sensitive -k 2*’. 3) Reads were then paired and those where both ends mapped to unique/unambiguous genome positions were kept. 4) The mapped read pair positions were allocated to specific RE1-RE1 restriction digest fragments and these were filtered to remove any aberrant molecular events (e.g. circularisation) and pairs that convey no spatial information; mostly re-ligation of adjacent fragments and pairs internal to the same RE1 fragment. 5) Read pairs were grouped according to whether they represent the same ligation event, i.e. between the same restriction fragment ends. Unsupported ligation events, represented by only a single read pair, were discarded while the remaining, supported ligations (i.e. with two or more read pairs) were simplified into single non-redundant contacts. 6) Promiscuous contacts, where an end is also involved in another, different contact, are excluded (given that this should generally not be possible in a haploid cell).

The general command parameters used for processing all the sequence data was:

nuc_process -i in_r1.fq in_r2.fq -g genome_mm10 -v -re1 MboI -s 50–5000

It should be noted that that MboI and DpnII restriction endoculeases are isoschizomers, and so this command is also appropriate for the Flyamer et al. [12] and Ramani et al. [14] data where DpnII was used. For Stevens et al. [9] the option -re2 AluI was also added, except in the case of the “Cell 3” sample. A selection of the output processing statistics from the individual cells were then aggregated for each experimental protocol, as presented in Table 1.

The contact maps for each data set were automatically generated using the NucProcess software, along with scores for identifying the genome content (ploidy) and data sets with condensed, mitotic chromosomes (see Lando et al. [11]). These scores were used to identify haploid cells that were likely in the G1 phase of the cell cycle, i.e. non-condensed and without a doubled genome content, indicative of G2. In practice, the Stevens et al. [9] and Flyamer et al. [12]. data sets did not require G1 phase selection as they were already selected/separated according to cell cycle. For the Nagano et al. [13] data sets 20 good G1 candidates were readily identified from the “Haploid3” subset. For the Ramani et al. [14] data there were too few contacts for individual cells to make an assessment of cell cycle stage, so no selection was made and the 500 most populous data sets were used for analysis.

The distribution of contact probability according to sequence separation (i.e. within the same chromosome) was plotted for aggregated data sets for each experimental protocol using the “*nuc_contact_probability*” program.

### Structure calculation

The best data set from each of the Flyamer et al. [12], Nagano et al. [13] and Stevens et al. [9] cells were identified by inspection of the sequence processing reports and contact maps. In each case we selected the cells with the most contacts that were likely to be in G1 phase, and also showed clear clustering of inter-chromosomal contacts and, where possible, low random/dispersed nose. The contact list for each cell (in the NCC text format, as output by NucProcess) was input to the NucDynamics program (https://github.com/tjs23/nuc_dynamics) and used to calculate 10 independent coordinate models for each genome, using the same, default parameters for each cell:

nuc_dynamics -m 10 -cpu 10 input.ncc -o output.n3d

Accordingly, the genome structures were calculated using a hierarchical simulated annealing procedure, which starts from random coordinates (in a sphere) and subsequently solves structures at ever higher resolution (8.0, 4.0, 2.0, 0.4, 0.2 Mb particle sizes) until a final resolution with 100 kb particles. It should be noted that as standard, to reduce isolated contact noise, the software discards any contacts that are deemed unsupported; where there is no other contact within 2 Mb of both ends.

For each single cell, the coordinates were output and the separate models were aligned with an iterative, weighted singular-value decomposition approach, as described in Stevens et al. [9] so that the all-particle RMDs values could be computed between the various different models that derive from each cell/protocol.

## Supplementary Material

KNCL_A_1438799_Suppl_Mat.zip

## Accession numbers

Datasets used in this study had already been deposited in the Gene Expression Omnibus (GEO) with the following accession numbers GSE80006^12^, GSE80280^9^, GSE84920^14^ and GSE94489^13^.
