# Supplementary material for: Calculation of 3D genome structures for comparison of chromosome conformation capture experiments with microscopy: An evaluation of single-cell Hi-C protocols
Source: Nucleus. 2018 Mar 8;9(1):190–201. doi: 10.1080/19491034.2018.1438799 (PMC5883084; doi:10.1080/19491034.2018.1438799)
Supplement: KNCL_A_1438799_Suppl_Mat.zip [file kncl-09-01-1438799-s001.zip › KNCL_A_1438799_Suppl_Mat/2017NUCLEUS0035R-f06-z-4c.pdf]

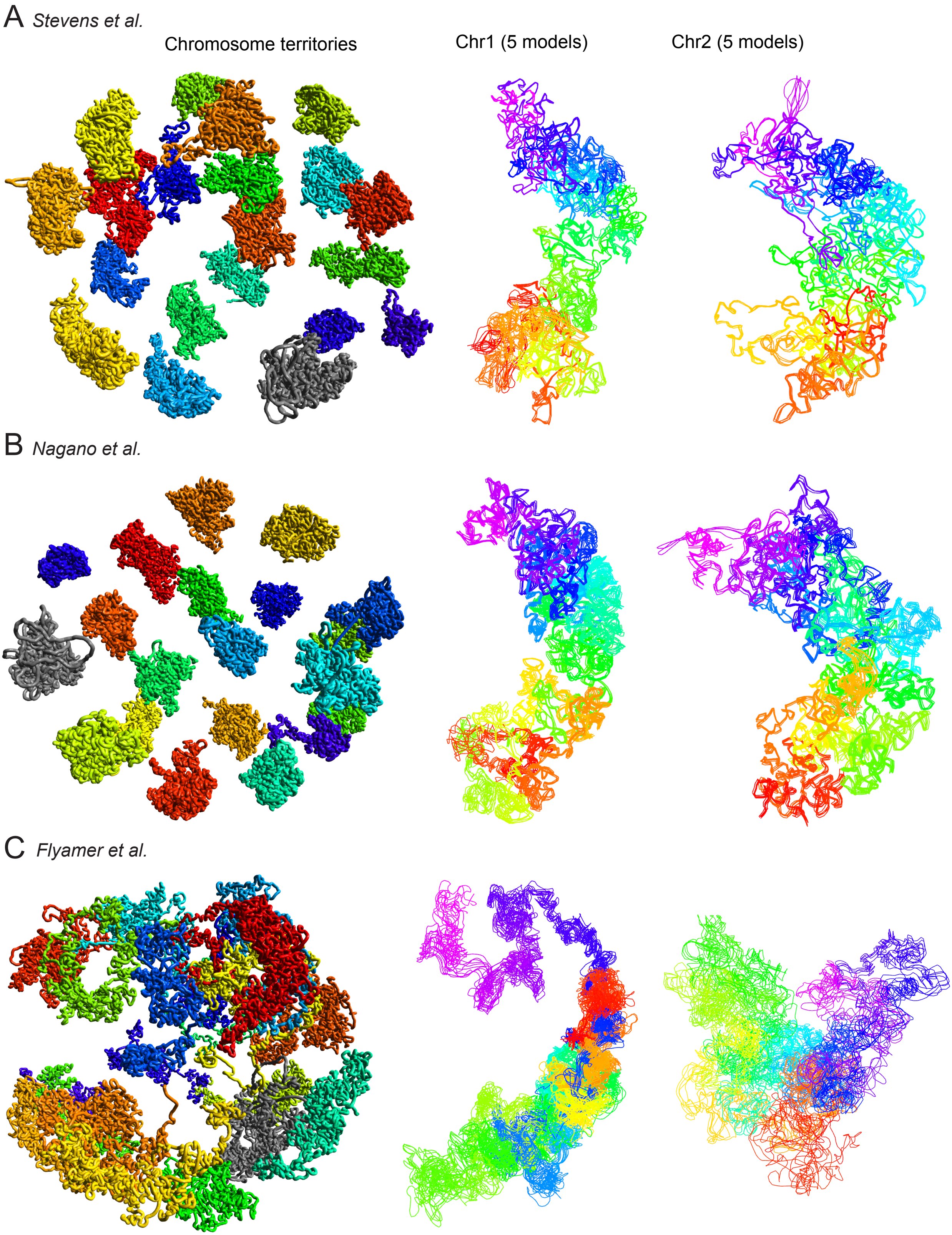

Figure S1. Further comparison of the 3D genome structures calculated from different single-cell Hi-C contact data-sets. The left hand panels show an exploded view of the structures shown in Fig. 5 where each of the 20 chromosomes is coloured differently. The right hand panels show five modelled conformations for the structures of chromosomes 1 and 2 where the backbone is coloured according to sequence position from red to blue (centromeric to telomeric end).
